# Antihypercholesterolemic effect of Cleome arabica L. on high cholesterol diet induced damage in rats

**DOI:** 10.17179/excli2015-169

**Published:** 2015-07-06

**Authors:** Noura Samout, Hafsia Bouzenna, Amani Ettaya, Abdelfattah Elfeki, Najla Hfaiedh

**Affiliations:** 1 Laboratory of Environmental Physiopathology, Valorization of Bioactive Molecules and Mathematical Modeling, Faculty of Sciences of Sfax, Road Soukra km 3.5- PB n° 1171-3000, Sfax, Tunisia; 2 Laboratory Animal Eco Physiology, Faculty of Sciences of Gafsa, Sidi Ahmed Zarrouk, 2112, Gafsa, Tunisia

**Keywords:** hypercholesterolemic rat, Cleome arabica, lipid peroxidation (TBARS), antioxidant enzymes

## Abstract

Dietary cholesterol is known to be one of the main risk factors that accelerate oxidation process leading to hypercholesterolemia and attendant cardiovascular diseases. The purpose of this study, carried out on adult male Wistar rats, was to evaluate the inhibitory effects of supplementation with aqueous of *Cleome arabica *leaf extract on hypercholesterolemia. After 3 months of treatment, animals were sacrificed by decapitation. Blood serum was obtained by centrifugation. Under our experimental conditions, administration of *Cleome arabica* leaf extract decreased the total cholesterol (TC), LDL-cholesterol (LDL-chol) and triglycerides (TG) levels by 27 %, 52 %, 37 %, respectively, and reduced SGOT SGPT, LDH and PAL levels in blood serum compared to untreated hypercholesterolemic rats. TBARS concentrations decreased by 21 % in liver, 22 % in heart and 30 % in kidney in a group of rats treated with cholesterol and *Cleome arabica* (Chol C.ar) compared to a Chol-treated group. The same treatment with *Cleome arabica *leaf extract increased superoxide dismutase and enhanced glutathione peroxidase activity. Catalase activity was found to increase in liver, heart and kidney by 17 %, 16 % and 23 %, respectively, in the C.ar Chol-treated group. The protective effect of *Cleome arabica* on hypercholesterolemia inducing oxidative stress in several organs was mainly attributed to antioxidant properties. The latter were due to the presence of phenolic acids and flavonoids shown by the obtained HPLC profiles.

## Introduction

Hypercholesterolemia is one of the major risk factors for cardiovascular diseases. It also causes oxidative stress resulting in increased lipid peroxidation in multiple organs (Cai et al., 2014[3]). Previous studies also indicate that hypercholesterolemia is causally linked to a significant increase in reactive oxygen species with concomitant decrease in thiol levels in heart tissues (Sudhahar et al., 2007[20]; Ouslimani et al., 2005[18]). Hypercholesterolemic diet leads to various spontaneous arterial vasoconstrictions including renal artery vasoconstriction associated with decreased glomerular filtration rate in normal rats (Campos et al., 1999[4]). Moreover, severe liver damage, perturbations of cholesterol metabolism and hyperglycemia have been associated with hypercholesterolemia. 

Over the past several years, a growing interest has been focused on dietary regimen and, more particularly, natural products because of their capacity to reduce toxicity as well as their protective effects on oxidative stress and related health problems including age-associated diseases and cancer.

In this respect, *Cleome arabica* has attracted a great deal of attention in recent studies conducting antioxidant-related research. This plant belongs to the family Capparaceae which has several species widely grown in tropical and subtropical regions of the world, more specifically in North Africa (Ladhari et al., 2013[12]). It has been investigated and shown to serve several important activities for medical purposes such as treatment of inflammation (Tsichritzis et al., 1993[21]), rheumatism (Ahmad et al.,1990[2]) and cytotoxicity (Nagaya et al.,1997[17]). *Cleome arabica* has been shown to scavenge free radical species and modulate lipid peroxidation levels by stimulating the defense system (Selloum et al.,1997[19]). *Cleome arabica* contains a large variety of antioxidant compounds such as syringic acid, vanillic acid, quercetin and kaempferol (Ladhari et al., 2013[12]).

The main objective of the present experimental study is to investigate whether a *Cleome arabica-*enriched dietary regimen exerts a preventive effect on hypercholesterolemia inducing hepatic, renal and cardiac toxicity.

Serum TC levels, LDL, HDL-cholesterol (HDL-chol), TG,SGOT, SGPT, LDH, PAL, urea, uric acid, creatinine, lipid peroxidation level and activities of antioxidant enzymes (catalase, superoxide-dismutase and glutathione-peroxidase) in liver, heart and kidney were measured.

A phytochemical study of the *Cleome arabica* leaf extract was carried out in order to identify some antioxidative substances. 

## Material and Methods

### Preparation of the aqueous Cleome arabica leaf extract

The leaves and stems of *Cleome arabica *were collected from the Gafsa region located in south-western Tunisia. They were dried in the shade at a research laboratory for one week then ground. Extraction of *Cleome arabica *was carried out by immersing 5 g of the plant in 100 ml distilled water for 5 to 10 min followed by filtration and gavage administration.

### Experimental rats and diets

Healthy adult male rats of the Wistar strain (n=28) weighing 100 ± 5 g were used for the present study. The animals were purchased from Central Pharmacy of Tunisia, Tunis, Tunisia. The animals were randomly divided into four groups of seven rats each. Each group was housed in a spacious indoor cage with a relative humidity of 60 % at room temperature of 24 °C. Before treatment, rats were allowed to adapt to their new environment for 7 days. The animals were kept under photoperiods (a 12 h light/dark cycle) for 3 months. [Note: All the experimental procedures were carried out in compliance with Guidelines on the Use of Living Animals in Scientific Investigations.] The rats were fed for 3 months as follows:

- Group 1: control group (T) fed with normal diet.
- Group 2: Chol group fed with 1 % cholesterol-enriched diet in combination with 0.5 % cholic acid(Chol).
- Group 3:C.ar group fed with normal diet + *Cleome arabica* (C.ar) lyophilized aqueous extract (0.5 %).
- Group 4: Chol C.ar group fed with 1 % cholesterol-enriched diet in combination with 0.5 % cholic acid + *Cleome arabica* (C.ar) lyophilized aqueous extract (0.5 %) (Chol+C.ar).

### Blood samples

After 3 months, rats from each group were rapidly sacrificed by decapitation in order to minimize the handling stress. Blood samples were collected from jugular vein in dried tubes and centrifuged at 1500 g for 15 min at 4 °C. Serum samples were collected for biochemical analyses to determine the TC rate, HDL-chol, LDL-chol and TG. Liver, heart and kidney were also quickly excised, cleaned of fat and stored at -80 °C until use.

### Extraction and analysis of phenolic compounds

2 g of the air-dried plant material were mixed with 10 ml of 80 %aqueous methanol (v/v) and gently agitated at room temperature for 10 min, followed by a centrifugation for 5 min at 12000 rpm. Another successive extraction with 0,5 ml of supernatant and 0,5 ml of acetone was conducted at room temperature for 30 min.

### HPLC analysis conditions

A Varian Prostar HPLC system equipped with a ProStar 230 ternary pump, a manual injector and a ProStar 330 diode array detector were used. The chromatographic analyses were performed on a 5 µm particle C-18 reversed-phase column (Varian, 250×4.6 mm). Final chromatographic conditions were a gradient elution made up of solvent A: 0.05 % acetic acid aqueous solution and solvent B: methanol. At t=0, the mobile phase consisted of 65 % A and 35 % B, and it changed with a linear gradient to 50 % A and 50 % B in 30 min. A change in the methanol under observation occurred during each 5-min interval until it reached 20 % A and 80 % B within a total duration of HPLC-based analysis of 40 min. The flow rate amounted to 1 ml/min with an injection volume of 20 µl at 25 °C. UV detection was performed at 365 nm for four flavonoids (luteolin, quercetin, kaempferol and isorhamnetin) and at 290 nm of phenolic acids. Identification of such compounds was carried out by comparing the retention time and mass spectra of the peaks in the injected sample extracts to those of HPLC standard compounds.

### Biochemical assays

The total cholesterol level, HDL, LDL-chol, TG as well as activities of glutamic-oxaloacetic transaminase (SGOT), glutamic-pyruvic transaminase (SGPT), alkaline phosphatase (PAL), lactate dehydrogenase (LDH), urea, uric acid and creatinine (Crea) were determined by kit methods (Spinreact).

In accordance with Yagi (1976[23]), the lipid peroxidation level was measured as thiobarbituric acid reactive substances (TBARS). In this study, 125 μl of supernatant (S1 liver, heart or kidney) were mixed with 175 μl of 20 % trichloroacetic acid containing 1 % butyl-hydroxytoluene and centrifuged (1000 x *g*, 10 min, 4 °C). Then, 200 μl of supernatant (S2) was mixed with 40 μl of HCl (0.6 M) and 160 μl of thiobarbituric acid (0.72 mM), and the mixture was heated at 80 °C for 10 min. Absorbance was measured at 530 nm. The amount of TBARS was calculated using an extinction coefficient of 156 mM^−1^ cm^−1^ and expressed in nmol/mg protein.

Superoxide dismutase (SOD) activity was determined by measuring its ability to inhibit the photoreduction of nitroblue tetrazolium (NBT) using Hara et al.,'s method (1972[8]). One unit of SOD represents the amount capable of inhibiting the photoreduction of NBT by 50 %. The activity was expressed as units/mg protein, at 25 °C.

Glutathione peroxidase (GPx) was determined by the method of Flohe and Gunzler (1984[7]). One unit of GPx was defined as oxidation by H_2_O_2_ of 1 µmol of reduced glutathione peroxide per min at a pH of 7 and a temperature of 25 °C.

Catalase (CAT) activity was measured according to Aebi (1984[1]). The reaction mixture (1 ml) contained 100 mM phosphate buffer (pH=7), 100 mM H_2_O_2_ and 20 μl (about 1-1.5 mg of protein) of liver, heart and kidney. H_2_O_2_ decomposition at 25 °C was followed by measuring the decrease in absorbance at 240 nm for 1 min. Enzyme activity was calculated using an extinction coefficient of 0.043 mM^−1^ cm^−1^ and expressed in international units (I.U.), i.e. in μmoles H_2_O_2_ destroyed/min/mg protein. Tissue extract protein content was determined according to Lowry's method (Lowry et al., 1951[15]) using bovine serum albumin as standard.

### Statistical analysis

Results are expressed as mean ± standard deviation (SD) and the statistical significance was assessed by Student's t-test. P < 0.05 was considered statistically significant.The limit of statistical significance was set at P < 0.05 between both the *Cleome arabica*-treated and untreated groups.

## Results

Body weight, food and cholesterol intake

In this study, as shown in Table 1(Tab. 1), the dietary cholesterol induced an increase in body weight compared to the control group (T) (P < 0.05) after a 3-month treatment.

Total cholesterol, LDL, HDL-chol and triglycerides in blood serum

The obtained results showed a significant increase in the total cholesterol, LDL-chol and triglycerides levels, and a significant decrease in HDL-chol in the hypercholesterolemic rats compared to controls. After three months of treatment with* Cleome arabica *leaf extract, these biomarkers were found to revert to almost control values (Table 1(Tab. 1)).

Serum markers of liver and kidney damage 

**Figure 1:**
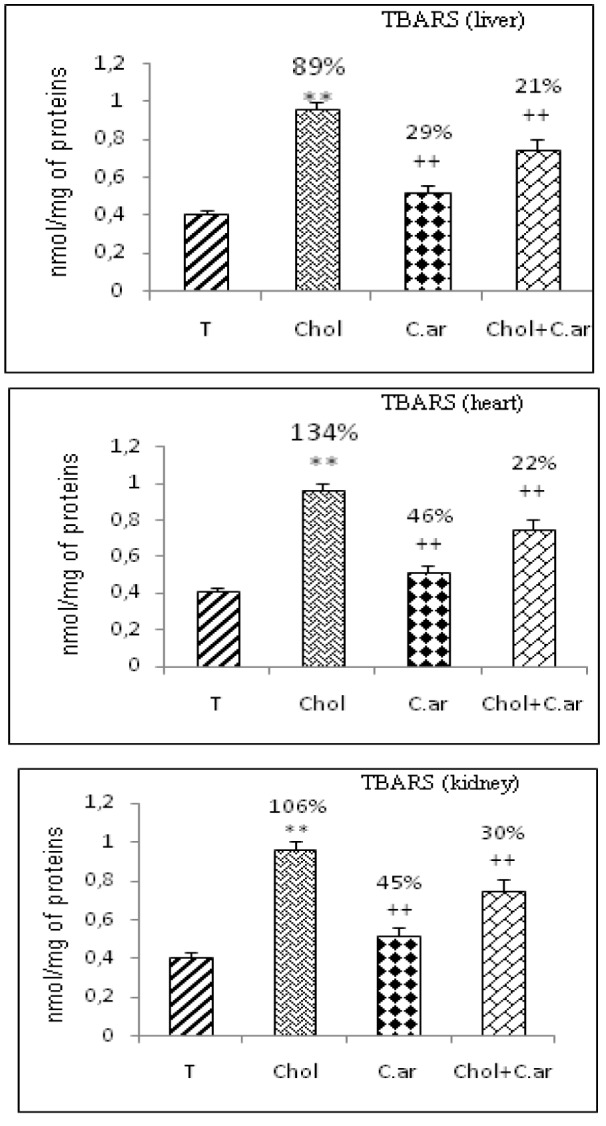
Lipid peroxidation level (expressed as TBARS, nmol/mg protein) in liver, heart and kidney after 3 months of treatment in controls (T), (Chol) cholesterol-treated rats, (Chol C.ar) cholesterol-treated rats supplemented with *Cleome arabica *leaf extract, (C.ar) rats supplemented with *Cleome arabica *leaf extract

**Figure 2:**
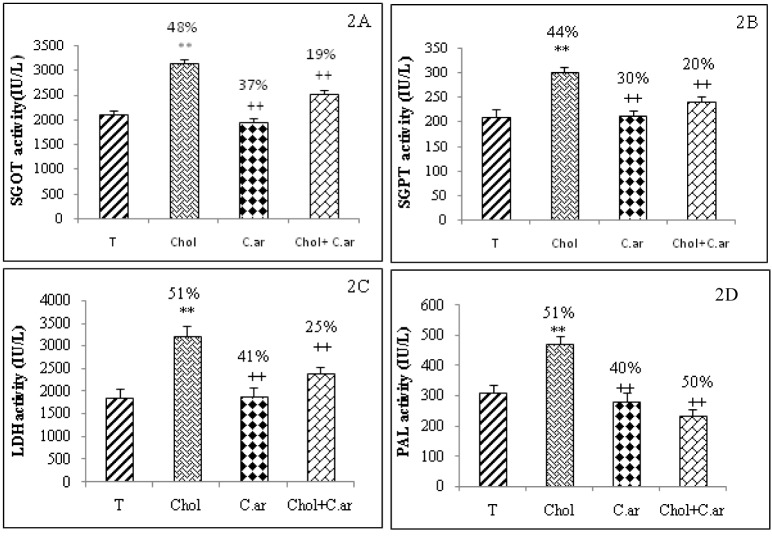
Effect of hypercholesterolemia and *Cleome arabica* on SGOT (2A), SGPT (2B), LDH (2C) and PAL (2D) activities. Values are mean ± SEM; n = 7 * Chol compared to control group (T): P < 0.05; ** P ≤ 0.01 compared to control group (T) ^+^ compared to Chol group: P < 0.05; ^++ ^P ≤ 0.01 compared to hypercholesterolemic group (Chol)

Glutamic-oxaloacetic transaminase (SGOT), glutamic-pyruvic transaminase (SGPT), alkalinephosphatase (ALP) and lactate dehydrogenase (LDH) were released into the blood when certain organs or tissues were injured. As shown in Figure 1(Fig. 1), these activities were significantly greater in hypercholesterolemic rats than in controls (T). Hypercholesterolemia also induced a clearly noticeable kidney damage due to a significant increase in urea, uric acid and creatinine levels in serum (Figure 2(Fig. 2)). All these biomarkers reverted to almost normal values in the case of *Cleome arabica*-treated hypercholesterolemic rats.

Oxidative damage 

**Figure 3:**
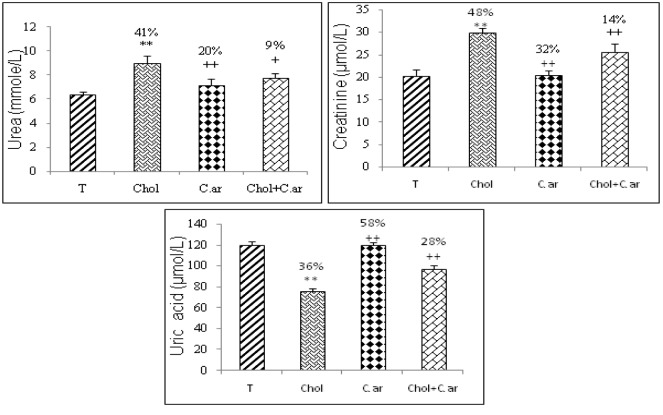
Effects of hypercholesterolemic diet, *Cleome arabica* on urea (mmol/L), creatinine (µmol/L) and uric acid (µmol/L) levels in blood. Values are the mean of 7 measurements ± SD. *P < 0.05 compared with control (T); **P < 0.01 compared with control ^+^ P < 0.05 compared with hypercholesterolemic rats (Chol); ^++^P < 0.01 compared with (Chol).

In the case of rats receiving dietary cholesterol, TBARS levels in hepatic, cardiac and renal tissues increased by 89 %, 134 % and 106 %, respectively, when compared to controls (Figure 3(Fig. 3)). The administration of *Cleome arabica* leaf extract resulted in a significant reduction in these TBARS levels.

Antioxidant activities

The enzyme activities that had a protective effect against ROS, i.e. CAT and GPx, were found to decrease by 26 %, 22 % in liver, 16 %, 21 % in heart and by 31 %, 41 % in kidney. Table 2(Tab. 2) shows that SOD activity decreased by 47 % in liver, 19 % in heart and by 26 % in kidney of hypercholesterolemic rats compared to controls. These changes, which revealed a failing defense against oxidative stress, were largely corrected in animals treated with *Cleome arabica*.

### HPLC analysis of Cleome arabica phenolic acids and flavonoids

**Figure 4:**
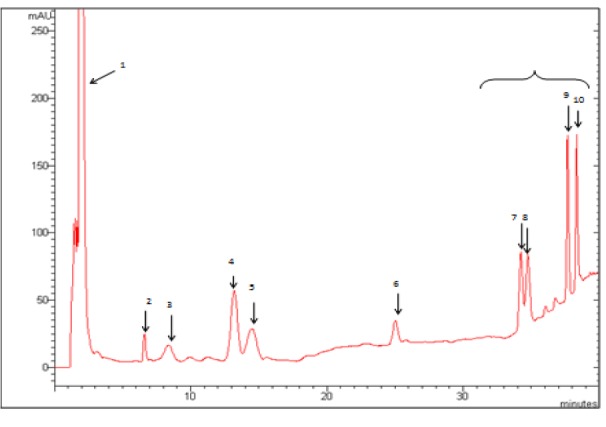
HPLC elution profile of phenolic acids (λ = 290 nm) from lyophilized aqueous extract of *Cleome arabica *

**Figure 5:**
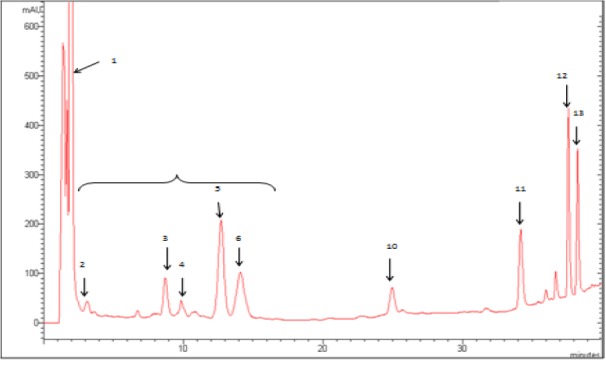
HPLC elution profile of flavonoids (λ = 365 nm) from lyophilized aqueous *Cleome arabica* leaf extract

As shown in Figure 4(Fig. 4), the HPLC analysis of *Cleome arabica* phenolic acids in 290 nm revealed the presence of phenolic acids. Three phenolic acids were identified in FPA: protocatechic, gallic, vanillic and syringic acids. The HPLC elution profile of flavonoids (Figure 5(Fig. 5)) also illustrated seven peaks of unknown compounds and four flavonoids in 365 nm: quercetin, luteolin, kaempferol, isorhamnetin. Phenolic acids and flavonoids contributed to the beneficial effect of *Cleome arabica *leaf extract *in vivo*.

Peaks (1) acetone peak, (2) protocatechic acid, (3) gallic acid, (4) 4-hydroxybenzoic acid, (5) vanillic acid, (6) syringic acid, (7, 8, 9 and 10) unknown compounds. The HPLC analyses were performed using a Varian ProStar HPLC equipped with a reverse phase C-18 column (Varian, 150 mm×4.6 mm, particle size 5 µm) in conjunction with a gradient elution (A, 0.05 % CH_3_COOH aqueous solution; B, methanol): t_0_ = 65 %A and 35 %B; 30 min = 50 %A and 50 % B; 40 min = 20 %A and 80 %B. The flow rate was 1 ml min^−1^ and the injection volume was 20 µl at 25 C.

Peaks (1) acetone peak, (2-6) unknown compounds, (10) quercetin, (11) luteolin, (12) kaempferol, (13) isorhamnetin. The HPLC analysis were performed using a Varian ProStar HPLC equipped with a reverse phase C-18 column (Varian, 150 mm×4.6 mm, particle size 5 µm) in conjunction with a gradient elution (A, 0.05 % CH_3_COOH aqueous solution; B, methanol): t_0_ = 65 %A and 35 %B; 30 min = 50 %A and 50 %B; 40 min = 20 %A and 80 % B.

## Discussion

The aim of this experimental study was to determine the protective effect of *Cleome arabica* on hypercholesterolemia in liver, heart and kidney. The biochemical parameters and activities of antioxidant enzymes were determined. The obtained results indicated that hypercholesterolemia led to statistically significant changes in biochemical parameters. It was found that an increase in total plasma cholesterol, LDL-chol, TG, SGOT, SGPT, LDH, PAL, urea and creatinine occurred with a decrease in HDL-chol and uric acid value compared to control group. Hypercholesterolemia caused elevated cholesterol level in serum as well as in multiple cells such as erythrocytes, platelets and endothelial cells. This increased cholesterol level was found to activate these cells and lead to enhanced production of reactive oxygen species (ROS) (Kamesh and Sumathi. 2012[11]). This mechanism induced alteration of vascular function, cell proliferation and apoptotic/neurotic process. Louala et al., (2013[14]) and Milagro et al. (2006[16]) reported that hypercholesterolemia increases lipid peroxidation in liver cells then stimulates lipogenesis in adipose tissue. Several studies have revealed that high cholesterol level can cause cardiac damage (Johnkennedy et al., 2011[10]). Markedly elevated level of serum cardiac marker enzymes such as SGOT and LDH in HDF-induced rats is due to peroxide formation generated by hypercholesterolemia in the form of ROS (Sudhahar et al., 2007[20]). Hypercholesterolemia also contributes to renal, glomerular, interstitial and vascular damage. In the present study, it was found that hypercholesterolemia did not only alter the liver, cardiac and renal biomarkers but also inhibited antioxidative defense mechanisms. In fact, the lipid peroxidation levels increased with decrease in SOD, CAT and GPx levels in heart, liver and kidney due to hypercholesterolemia. This led to aggravation of oxidative stress by reducing cellular glutathione levels, as observed in previous studies (Cai et al., 2014[3]).

Findings reported in several studies suggest that hypercholesterolemia-induced organ damage is probably associated with the accumulation of reactive oxygen species (Li et al., 2014[13]). Other findings relate hypercholesterolemia-induced damage in tissues to overproduction of free radicals and ROS resulting in enhanced lipid peroxidation, damage to DNA, protein degradation, and exhaustion of the antioxidative defense systems (Kamesh and Sumathi, 2012[11]).

Under our experimental conditions, *Cleome arabica* leaf extract administered by gavage corrected all the investigated parameters that had shifted to pathological values due to hypercholesterolemia. The decrease in TC, LDL-chol and TG levels in serum by 27 %,52 %, 37 %, respectively, compared to untreated hypercholesterolemic rats could be explained by the regulation of lipid metabolism. As has already been reported in previous research, *Cleome arabica* has an antioxidant activity owing to the presence of its phenolics and flavonoid compounds. Moreover, *Cleome arabica *protected liver, heart and kidney functions. It enabled reversion of these major organs' dysfunction indices such as SGOT, SGPT, LDH, PAL, urea, uric acid and creatinine activities almost back to normal values. 

A similar result was obtained by supplementation with certain dietary antioxidants rich in polyphenols and flavonoids extracted from *Cleome arabica* (Djeridane et al., 2010[5]). Indeed, flavonoids can increase HDL-chol and decrease LDL cholesterol oxidation. The defense mechanism against hypercholesterolemia consists of both antioxidants synthesized in the tissues and exogenous antioxidants supplied with diet. Interestingly, our results have demonstrated that *Cleome arabica* exerts a beneficial effect on body weight of hypercholesterolemic rats.This inhibitory activity by *Cleome arabica *leaf extract against oxidative stress could be explained by the presence of polyphenols and flavonoids in *Cleome arabica *leaf extract. This result is in agreement with previous data reporting that *Cleome arabica *leaf extract contains various flavonoids such as luteolin, kaempferol and isorhamnetin (Ladhari et al., 2013[12]). Actually, flavonoids existing in *Cleome arabica* leaf extract reduced lipid peroxidation and enhanced the expression of intracellular endogenous antioxidants such as SOD, GPx and catalase by maintaining their activities at a higher level than cholesterol-treated rats. This is due to the ability of such enzymes to scavenge free radicals, as confirmed by Cai et al. (2014[3]). Furthermore, the polyphenols and flavonoids present in *Cleome arabica *leaf extract can be considered as a highly effective antioxidant able to protect biological systems against the oxidative stress known as an important pathophysiological factor for a variety of diseases including hypercholesterolemia, cancer, diabetes, cardiovascular disorders and rheumatoid arthritis (Unnikrishnan et al., 2014[22]; Duchnowicz et al., 2012[6]).

Under our experimental conditions, *Cleome arabica* was found to be a valuable source of natural health-promoting antioxidants and ingredients such as phenolic acids (protocatechic acid, gallic acid, 4-hydroxybenzoic acid, vanillic acid, syringic acid and flavonoids (quercetin, luteolin, kaempferol, isorhamnetin). A number of studies have highlighted the relation between the chemical structure of phenolic acids and their ability to reduce free radicals by the release of a proton from the hydroxyl group against oxidative stress inducing damage in multiple tissues (Hfaiedh et al., 2014[9]).

In conclusion, the strong therapeutic effect and potential of *Cleome arabica* are arguably associated with its potent hypocholesterolemic properties. *Cleome arabica *was found to be able to significantly achieve almost the same degree of improvement in cholesterol-related pathological conditions in rat liver, heart and kidney.

## Acknowledgements

This research was funded by the Tunisian Ministry of Higher Education and Scientific Research through Integrated Physiology Laboratory: Faculty of Sciences of Gafsa. We would like to express our special thanks to l'Unité des Services Communs in findings in Faculty of Sciences of Gafsa, Tunisia, for technical support. The authors would like also to express their sincere thanks to Zied Tlili for his careful editing of the manuscript.

## Competing interests

The authors declare that they have no competing interests.

## Figures and Tables

**Table 1:**
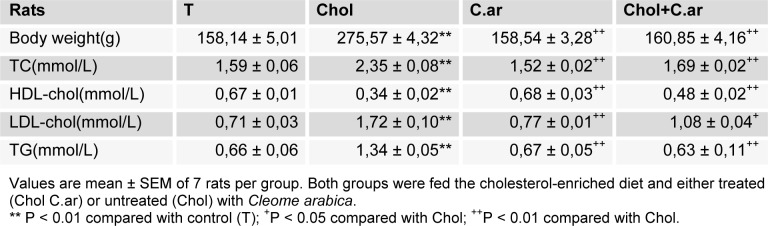
Body weight, TC, HDL, LDL-chol and TG in Control (T) and experimental rats after 3 months of treatment

**Table 2:**
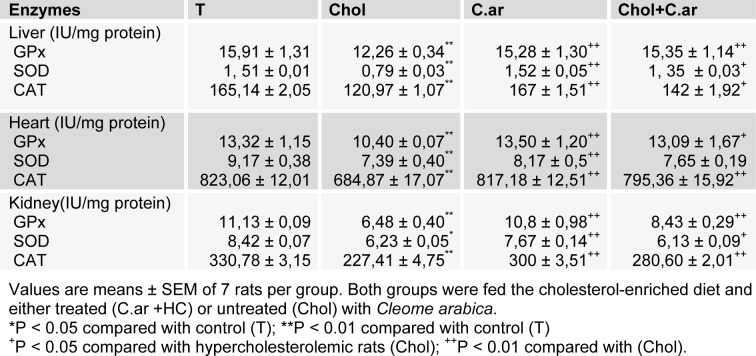
Antioxidant enzyme activity in hepatic, cardiac and renal tissues
